# The Fitting and Furnishing of Wards and Operating Theatres

**Published:** 1906-10-13

**Authors:** 


### THE FITTING AND FURNISHING OF WARDS AND OPERATING THEATRES.

### BEDSTEADS, LAUNDRIES, AND KITCHENS.

In the modern hospital all bedsteads are of iron, the posts being of steel-tube, painted with stove-fired enamel, and all are provided with spring mattresses. The bedsteads are arranged for the convenience of the surgeon and the nurse, and also to afford the greatest possible comfort to the patient. The head of the bedstead is usually made removeable, so that the surgeon or anæsthetist can get behind the head of the patient. The body of the bedstead is made, in some forms, so that the portion near the head can be raised, and for epileptic patients the bedsteads are enclosed, very much like a child's crib, the side being arranged to let down when required. For maternity wards children's cradles made of strong steel wire also black enamelled, are arranged to be suspended from hooks attached to uprights fixed at the foot of the bedstead. One of the most perfect and complete bedsteads for hospital purposes is that in use at Guy's Hospital, which Sir Cooper Perry has made so practically efficient as to be almost if not quite unique. The Lawson-Tait bedstead is very largely employed, but the "Wood" bedstead made by the Longford Wire Company of Warrington is also used in many hospitals. Whitfield's Bedsteads, Limited, of Birmingham, have successfully attacked the problem of an easily moving noiseless bedstead. The castors of their bedsteads made for St. Thomas's Hospital have 4-inch rubber tyres, and the fork to which the castor is attached is arranged by a simple contrivance, but one that requires careful fitting, to swivel round the leg of the bedstead, so that no matter what obstacle is met with in transporting a patient there are no jerks.

#### Laundries.

The machinery employed in the laundry has been already described in the columns of The Hospital. Many of the large hospitals now have their own laundry, in which they deal with their own clothes, and also with those of some of the hospitals who have not their own laundry. There are two important features in connection with laundries, the condition of the atmosphere and of the floor. It is absolutely necessary to use steam in the modern laundry for many purposes, in the washing machines, and often in the ironing apparatus. The tendency therefore is for the laundry atmosphere to be a steamy one. Laundries also are places where sloppiness more or less necessarily abounds. But for the health of the employés the steamy atmosphere must be got rid of. In the laundry of the Victoria Hospital at the London Docks which the writer visited, these objects appeared to be fully accomplished. The atmosphere is not steamy, and the floor is not sloppy. Gutters are provided in the floor of the laundry, lined with glazed tiles, and leading to glazed pipes, the gutters being covered at the floor level by iron gratings, the result being that the floor is kept dry. The floor itself is of asphalt. The ventilation of this laundry is by two fans exhausting from the sides of the roof. The machinery of the laundry is driven by a steam engine, the exhaust steam being employed for heating the drying closets, and afterwards for heating the water for the laundry itself. A portion of the air from the laundry is carried through the drying closets, and carried out by the exhaust fan which creates the drying air current above the drying closets. Another important point in connection with laundries is the careful guarding of the machinery. It is absolutely necessary for driving the apparatus that cog-wheels of various forms shall be employed, and these should be so enclosed that even the most careless attendant cannot easily get her fingers in. A most important machine in this respect is the callender, the large machine, consisting of a polished steel drum lying in a semi-cylindrical iron bed, the bed and the drum both being heated by steam, or by gas, and the large articles, such as sheets quilts, blankets, etc., being placed in position by attendants on one side, and removed after passing between the drum and its bed on the other side. The whole machine is necessarily at a high temperature, and when the work is being put through quickly there is a danger, if proper guards are not provided, of the fingers of the attendants following the articles into the space between the roller and the bed. At the Victoria Hospital at the London Docks every machine is fully guarded in this respect, and the superintendent informed the writer that the total cost of washing worked out at 0.98d. per piece, 6,000 pieces a week being handled. Another important point in connection with the laundries is, if clothes are to last, the wiles of the manufacturers of various substances warranted to lessen the time of washing—and to produce very white clothes must be avoided. Most of them rot the material, though many of them produce a very white effect for the time. Always have any of them that are offered analysed.

### Kitchens.

The kitchens of the great hospitals are nearly always on one of the upper floors. Steam, gas, and anthracite coal are employed in cooking. For vegetables and for a number of the joints, poultry, etc., steam ovens are employed. The steam oven is practically a box of iron with a lining also of iron, the space between the outside and the lining being employed to distribute the steam. There are the usual shelves in the ovens made in the form of racks, and on the racks are placed trays of galvanised iron, in which the substances to be cooked, the joints, potatoes, etc., are placed. Steam is brought to the oven at a very low pressure, and is allowed to permeate all through the produce, the water formed by its condensation being drained off. For boiling vegetables also large coppers are employed, in which the heat is delivered to the produce by steam passing through a lining similar to that of the oven. The covers of the coppers are arranged with pulleys and balance weights overhead, so as to be easily lifted. There are also large hot plates standing in convenient positions in the kitchen, heated by gas or by the gases formed by the combustion of coal in a furnace attached to them. There are the usual large cooking ranges with ovens, etc., and stoves. There are also hot cupboards heated in various ways, and all of the apparatus in which fuel is employed are arranged—in some cases to stand against the wall, with a large canopy above keeping all the fumes to the chimney, and in other cases are fixed in the middle of the floor, the flue being taken down into the fl[illegible]r, and thence to a chimney in the ordinary way.

